# ICESat-2 river surface slope (IRIS): A global reach-scale water surface slope dataset

**DOI:** 10.1038/s41597-023-02215-x

**Published:** 2023-06-06

**Authors:** Daniel Scherer, Christian Schwatke, Denise Dettmering, Florian Seitz

**Affiliations:** Deutsches Geodätisches Forschungsinstitut der Technischen Universität München (DGFI-TUM), TUM School of Engineering and Design, Department of Aerospace and Geodesy, Munich, Germany

**Keywords:** Hydrology, Hydrology, Geomorphology

## Abstract

The global reach-scale “ICESat-2 River Surface Slope” (IRIS) dataset comprises average and extreme water surface slopes (WSS) derived from ICESat-2 observations between October 2018 and August 2022 as a supplement to 121,583 reaches from the “SWOT Mission River Database” (SWORD). To gain full advantage of ICESat-2’s unique measurement geometry with six parallel lidar beams, the WSS is determined across pairs of beams or along individual beams, depending on the intersection angle of spacecraft orbit and river centerline. Combining both approaches maximizes spatial and temporal coverage. IRIS can be used to research river dynamics, estimate river discharge, and correct water level time series from satellite altimetry for shifting ground tracks. Additionally, by referencing SWORD as a common database, IRIS may be used in combination with observations from the recently launched SWOT mission.

## Background & Summary

The water surface slope (WSS) is a fundamental parameter for calculating river discharge, one of the Essential Climate Variables (ECVs) as defined by the Global Climate Observing System^1^. River discharge critically contributes to the characterization of the Earth’s hydrological cycle and climate and, thus, its determination on a global scale is of great scientific relevance. Additionally, correcting water surface elevation (WSE) observations from satellite altimetry for WSS can significantly improve the accuracy of the resulting water level time series^2,3^. Depending on the river’s morphology, regulation, bed material, and basin size, the WSS can be highly variable in both space and time^4^.

Various methods exist to measure WSS based on field surveys, gauges, airborne sensors, or satellites. However, most of them face difficulties in capturing the temporal and/or spatial variability of WSS at global scale. Although they are very accurate, field surveys and airborne campaigns can only cover relatively small study areas within a short period of time because of the high human and financial effort. Gauge records are usually available over long periods at a high sampling rate, but their suitability to derive WSS is limited to free-flowing river segments covered by multiple gauges. Due to the small number of free-flowing rivers in developed areas^5^ and the lack of gauging stations in remote areas, global WSS coverage with gauges cannot be achieved. In contrast, radar satellite altimetry provides a globally distributed network of so-called virtual stations, but at the same time lacks simultaneous observations over short distances and provides much fewer measurements compared to gauges^6^. Additionally, the distribution of virtual stations along a river is irregular, so that radar satellite altimetry cannot be used to derive a globally homogeneous WSS dataset. Another space-based technique is the use of digital elevation models (DEM) such as the “Shuttle Radar Topography Mission” (SRTM) or the “Advanced Spaceborne Thermal Emission and Reflection Radiometer” (ASTER) data, which provide spatially continuous elevation measurements within the boundaries of the spacecrafts’ orbits. The elevation accuracy of the DEM data, however, is low, which leads to errors when deriving WSS for short reach lengths and narrow rivers^7,8^. In addition, also the temporal resolution of DEM data is low, if the models are time-dependent at all. Just recently (December 2022), the “Surface Water and Ocean Topography” (SWOT) satellite was launched, targeting a WSS accuracy of 17 mm/km^9^. It was demonstrated that 90% of the sensor’s slope errors are in the desired range^10^, but SWOT observations are not yet available. In contrast, the unique measurement geometry of the “Ice, Cloud, and Land Elevation Satellite 2” (ICESat-2) with six parallel laser beams enables instantaneous and highly accurate WSS observations since its launch in September 2018. Due to its dense ground track pattern, ICESat-2 is well suited for global studies of the Earth’s hydrosphere^11^. ICESat-2 WSE observations have already been used to derive WSS in small study areas^2,12^, but not at the global scale. WSS datasets at the global scale so far exist only on the basis of DEM data such as the “Global River-Slope” (GloRS^13^) or as part of the “SWOT Mission River Database” (SWORD^14^).

In a previous study^2^, we developed an approach to derive reach-scale WSS from ICESat-2 observations. The approach was applied to 815 reaches in Europe and North America where sufficient validation data was available. For 89% of those reaches, the approach could be used to estimate WSS with a median absolute error of 23 mm/km, almost complying with the SWOT requirements of 17 mm/km. For the remaining studied reaches, there were no or not sufficient observations from ICESat-2. In order to create the global “ICESat-2 River Surface Slope” (IRIS^15^) dataset, we applied our approach^2^ to all reaches defined within SWORD. By referencing SWORD, IRIS can be easily compared or combined with SWOT mission observations as they become available. IRIS is the first WSS dataset with global coverage based on ICESat-2 observations. In this paper, we briefly review the materials and methods and present the resulting dataset.

## Methods

**Fig. 1:**
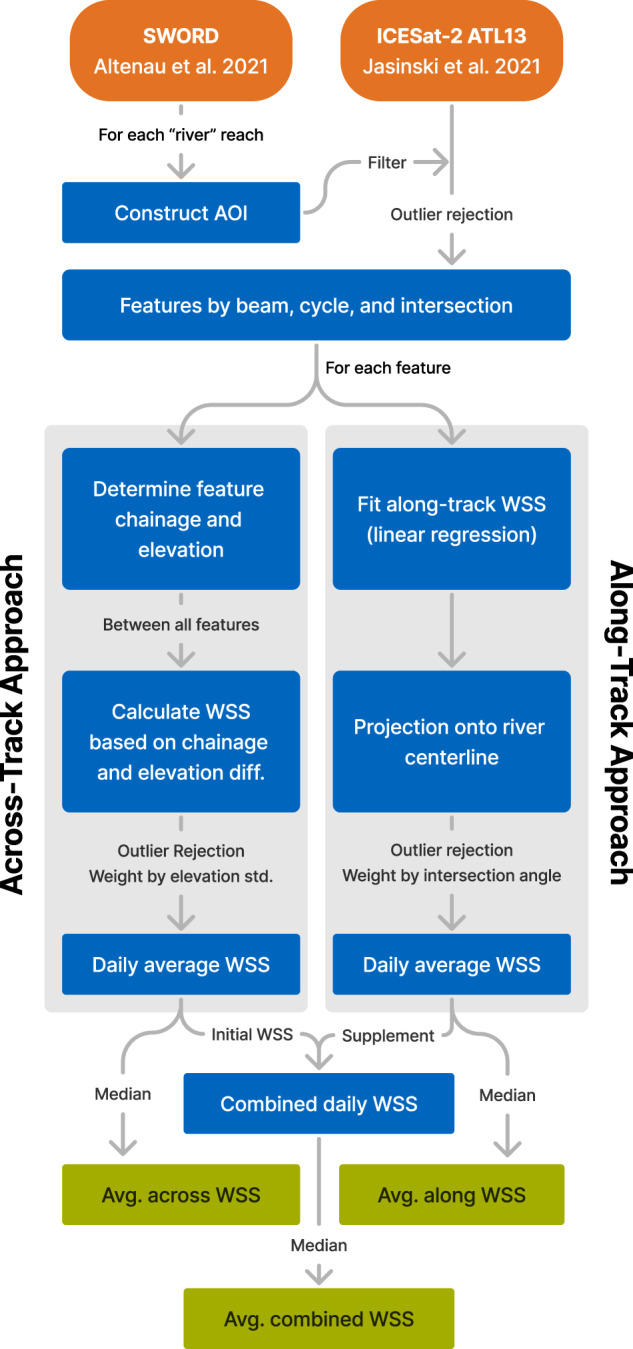
Processing strategy for the computation of the IRIS water surface slope (WSS) data.

Except for minor differences in preprocessing (more details below), the methodology used to derive the global “ICESat-2 River Surface Slope” (IRIS, Version v1^15^) dataset follows our published approach^2^, which has already been applied to derive and validate a regional dataset (Version v0^16^). Figure 1 shows a flowchart of the main steps in the processing of the IRIS dataset. The approach combines two different methods that are applicable depending on the intersection angle between the satellite orbit and the river: If ICESat-2 crosses a river reach nearly perpendicularly, the across-track approach calculates the WSS between the crossings of the sensor’s multiple beams. Otherwise, if satellite orbit and river are nearly parallel, the along-track approach calculates the WSS directly from the continuous water level observations along a single intersecting beam. The WSS within IRIS is defined as positive for a decreasing water surface elevation (WSE) in downstream direction. Table 1 lists the required input data. Besides the fundamental ICESat-2 and SWORD data, no auxiliary inputs are required. Version v1 of IRIS comprises ICESat-2 ATL13 version 5^17^ data from cycles 1 to 16 (October 2018 to August 2022) and uses SWORD version v2^14,18^. In the following, we briefly describe the materials and relevant processing steps. For a more detailed description, we refer the reader to our previous publication^2^.

**Table 1:** Input data for the IRIS dataset.

Dataset	Variable	Description
SWOT Mission River Database (SWORD^14,18^)		
	reach_id	ID of each reach (Used as key and to identify the reach type)
	centerline	Reach centerline shapefile geometry (Used to construct the reach AOI and measure the chainage)
	width	Average reach width (Used to construct the reach AOI)
ATLAS/ICESat-2 L3A Along Track Inland Surface Water Data (ATL13^17^)		
	ht_water_surf	Water surface height per short segment with reference to WGS84 ellipsoid
	cloud_flag_asr_atl09	Cloud probability using Apparent Surface Reflectance (ASR).
	snow_ice_atl09	NOAA snow/ice flag.

### SWOT mission river database (SWORD)

Version v1 of IRIS is designed as a supplement to version v2 of the “SWOT Mission River Database” (SWORD^14,18^), which contains high-resolution (30 m) river centerline geometries and river widths from the “Global River Widths from Landsat” (GRWL^19^) dataset segmented into reaches approximately every 10 km and topologically ordered. The direction of flow can be inferred from the reach identifiers, which increase upstream. The reaches are segmented at natural and artificial river obstructions such as dams and waterfalls, or anomalies like basin boundaries and tributary junctions. Therefore, we assume the WSS within each reach to be reasonably homogeneous. Each SWORD reach has an assigned type, and only reaches of type “*river*” or “*lake on river*” are processed for IRIS. SWORD also contains WSE and WSS data from MERIT Hydro^20^ which is derived from the multi-error-removed improved-terrain (MERIT) DEM^21^ based on SRTM. We use the SWORD WSS for comparison with IRIS. IRIS uses the SWORD reach identifier as a key so that both datasets can be used together.

In the first step of preprocessing, we buffer each SWORD reach’s centerline geometry by its average width to construct a polygon that defines the area of interest (AOI) for further processing. The resulting AOI is thus twice as wide as the average SWORD reach’s width to account for any significant temporal and spatial variability (e.g., on braided rivers). Note, that for the creation of the regional dataset (version v0), the AOIs were even wider by four times the width’s standard deviation^2^. We reduced the AOI size for version v1 because we observed a significant number of AOIs overlapping with adjacent water bodies on the global scale. In this way, some input data from ICESat-2 (see below) might be lost, but we also reduce the number of outliers.

### Ice, cloud, and land elevation satellite 2 (ICESat-2)

Each reach AOI is used to spatially filter WSE measurements from the “ATLAS/ICESat-2 L3A Along Track Inland Surface Water Data” (ATL13, Version 5^17^) dataset provided by the “National Snow & ICE Data Center” (NSIDC). These measurements are taken by ICESat-2’s photon-counting lidar sensor “Advanced Topographic Laser Altimeter System” (ATLAS) which determines the travel time of an emitted photon to the Earth and back to the sensor along three pairs of beams at a pulse rate of 10 kHz (i.e., one pulse every 0.7 m) and a footprint of approximately 17 m in diameter^22^. However, depending on water and atmospheric conditions, the sensor can detect only a maximum of 2.9 photons per meter over inland waters^23^. Each pair of beams consists of a high energy (175 ± 17 J) and a low energy (45 ± 5 J) beam. The energy of the beams used to estimate the WSS has no significant influence on the WSS accuracy^2^. The spatial resolution is relatively high compared to other repeat-orbit satellite altimetry missions because the 91-day repeat orbit with an inclination of 92 degrees and changing off-nadir pointings over a two-year period results in a track density of 2 km^24^. Version v1 of IRIS is based on ATL13 data from ICESat-2’s cycles 1 to 16 (October 2018 to August 2022). ATL13 does not contain photon-level observations but representative values over short segments of 75 to 100 consecutive received photons above inland water bodies. These short segments have an along-track length of 30 to several hundred meters, depending on the number of received signal photons per pulse^23^. We use the mean water surface height parameter (“*ht_water_surf*”) with reference to the WGS84 ellipsoid and apply the EIGEN-6C4 geoid^25^, which, unlike the EGM2008 geoid used for the ATL13 orthometric heights, also includes measurements from the GOCE mission. Additionally, we use the “*cloud_flag_asr_atl09*” and “*snow_ice_atl09*” (new in version v1 compared to v0) parameters to identify and reject outliers caused by clouds and ice coverage. All remaining ATL13 observations within the respective AOI are grouped by beam, cycle, and individual river intersection into so-called features (3D-geometries containing points of common properties). For each feature *i*, the chainage value *x*_*i*_ of its intersection with the river centerline or otherwise of the nearest point of the centerline is determined. We detect further outliers within each feature by calculating the absolute deviation around the median (ADM) within a rolling window and a linear support vector regression (SVR), similar to the approach applied in DGFI-TUM’s “Database for Hydrological Time Series over Inland Waters” (DAHITI^26^). Observations deviating more than 5 cm from the SVR or the respective median are rejected. If a feature contains a gap larger than 500 m, it is split at this gap, and only the largest cluster is processed further.

### Estimation of across-track WSS

For each feature *i*, we calculate the average elevation *h*_*i*_ of all valid ATL13 observations, weighted by their inverse distance to the river centerline. Then, the instantaneous WSS between *i* and every other feature *j* observed at the same date and within the same reach can be calculated as follows:

$$WSS(i,j)=\frac{{h}_{i}-{h}_{j}}{{x}_{i}-{x}_{j}}$$

Pairs of features with $\left|{x}_{i}-{x}_{j}\right| < 1$ km are not considered, and negative WSS estimates are viewed as outliers and rejected. Multiple instantaneous WSS observations of identical dates are averaged, weighted by the inverse sum of WSE standard deviations in both respective features, to get a reach-scale across-track WSS time series with daily temporal resolution.

### Estimation of along-track WSS

Taking advantage of the high spatial resolution, precision, and accuracy of ATLAS, the along-track WSS (tan *β*) can be estimated by fitting a linear regression to the ATL13 WSE observations and their position along the track within a single feature. However, tan *β* only represents the WSS along the beam ground track which is not fully parallel to the river centerline. This results in an erroneous WSS. Therefore, tan *β* is projected onto the river centerline tangent vector $\overrightarrow{c}$ to obtain an undistorted WSS along the river:

$$WSS=\frac{\parallel \overrightarrow{b}\parallel tan\beta }{\parallel \overrightarrow{b}{\rm{{\prime} }}\parallel }{\rm{s}}{\rm{g}}{\rm{n}}(\overrightarrow{b}\cdot \overrightarrow{c})\,{\rm{w}}{\rm{i}}{\rm{t}}{\rm{h}}\,\overrightarrow{b}{\rm{{\prime} }}=\frac{\overrightarrow{b}\cdot \overrightarrow{c}}{{\parallel \overrightarrow{c}\parallel }^{2}}\overrightarrow{c},$$

where $\overrightarrow{b}$ is the vector of the feature’s beam ground track segment. As above, negative WSS are rejected. In addition, an angle-dependent outlier threshold is applied to the confidence interval (CI) of tan *β*. The smaller the angle between $\overrightarrow{c}$ and $\overrightarrow{b}$, the higher the allowed CI, with a maximum angle of 65° and a maximum CI of 300 mm/km. These constraints were determined empirically by comparison with *in-situ* data^2^. We obtain a reach-scale along-track WSS time series by averaging the instantaneous results from identical dates weighted by the inverse of the angle between $\overrightarrow{c}$ and $\overrightarrow{b}$.

### Combined estimation of WSS

To increase the overall spatial and temporal coverage, we combine both methods. Depending on the intersection angle, only one of them may provide a WSS result. Thus, in the combination the reach-scale daily averaged across- and along-track WSS time series are merged, with the across-track results being preferred in the case of overlapping dates as this is the more accurate and robust approach^2^.

## Data Records

The global “ICESat-2 River Surface Slope” (IRIS^15^) dataset is available at Zenodo. IRIS is stored in a single NetCDF4 file, which is structured in a single group containing the variables listed in Table 2. IRIS can be used as a supplement to SWORD by joining the datasets via the “*reach_id* ” key. Otherwise, the “*lon*” and “*lat*” variables give the approximate centroid of the reach. All other variables are provided separately for each method. The “*[across/along/combined]_ flag*” variable indicates the availability of data from the respective method.

**Table 2:** Contents of the resulting IRIS dataset.

Variable Name	Unit	Description
reach_id		The SWORD reach identifier
lon	degrees east	Approx. centroid longitude of the SWORD reach
lat	degrees north	Approx. centroid latitude of the SWORD reach
across_flag		Flags indicating whether ICESat-2 [across/along/combined] slope is available (1) for the reach or not (0)
along_flag		
combined_flag		
avg_across_slope	mm/km	Average (median) ICESat-2 [across/along/combined] slope for the reach
avg_along_slope		
avg_combined_slope		
min_across_slope	mm/km	Minimum ICESat-2 [across/along/combined] slope for the reach
min_along_slope		
min_combined_slope		
max_across_slope	mm/km	Maximum ICESat-2 [across/along/combined] slope for the reach
max_along_slope		
max_combined_slope		
std_across_slope	mm/km	ICESat-2 [across/along/combined] slope standard deviation for the reach
std_along_slope		
std_combined_slope		
n_across_slope	days	Number of days with ICESat-2 [across/along/combined] slope observations for the reach
n_along_slope		
n_combined_slope		
min_date_across_slope	days since 2000-01-01	First date of ICESat-2 [across/along/combined] slope observations for the reach
min_date_along_slope		
min_date_combined_slope		
max_date_across_slope	days since 2000-01-01	Latest date of ICESat-2 [across/along/combined] slope observations for the reach
max_date_along_slope		
max_date_combined_slope		

**Fig. 2:**
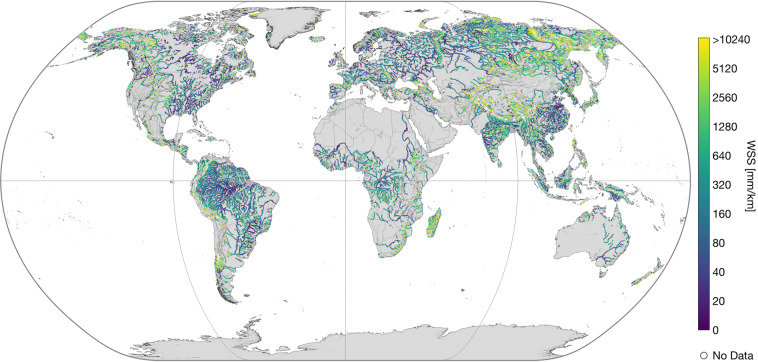
Averaged combined water surface slope (WSS).

**Fig. 3:**
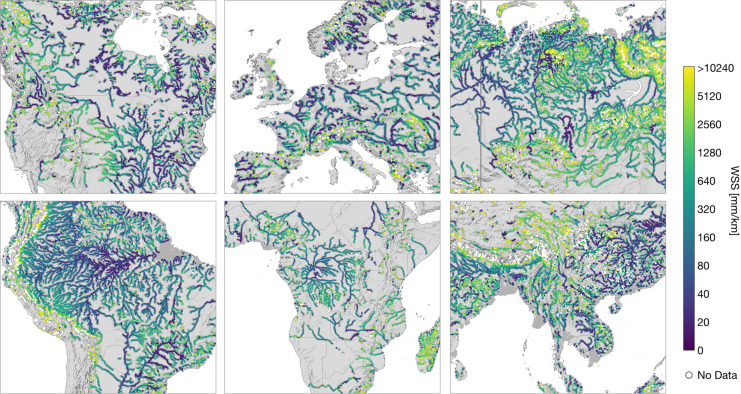
Detailed views of the averaged combined WSS for North America, Europe, and Siberia (upper f.l.t.r.), South America, Central Africa, and East Asia (lower f.l.t.r.).

**Fig. 4:**
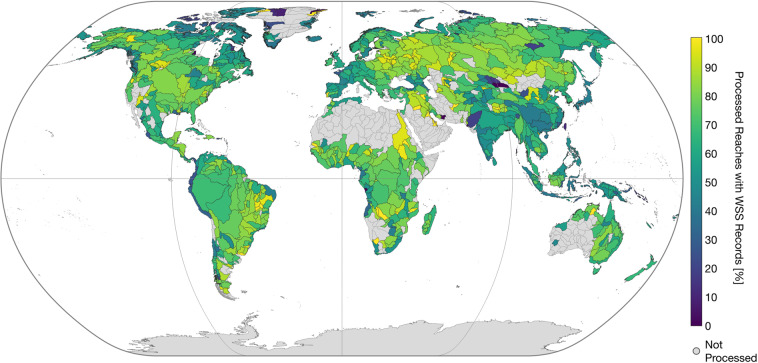
Percentage of processed reaches with Water Surface Slope (WSS) results per Pfaffstetter level 4 basin.

The main content of IRIS is the reach-scale median WSS from the combined approach (“*avg_combined_slope*”) shown in Fig. 2. More detailed views on specific regions are presented in Fig. 3. In dark grey, both figures display reaches that were not processed due to the SWORD type flag filter, such as ghost reaches or reaches with unreliable topography. Reaches that have been processed but do not contain valid results are shown in white. Overall, 178,659 (74.1%) of the total 241,107 reaches in the SWORD dataset pass the type flag filter (types “*river*” or “*lake on river*”) and are further processed. The approach yields results for 121,583 reaches which corresponds to a coverage of 68.1% of the processed SWORD reaches. Figure 4 shows the ratio of processed reaches with WSS results per river basin (Pfaffstetter level 4^27^).

**Fig. 5:**
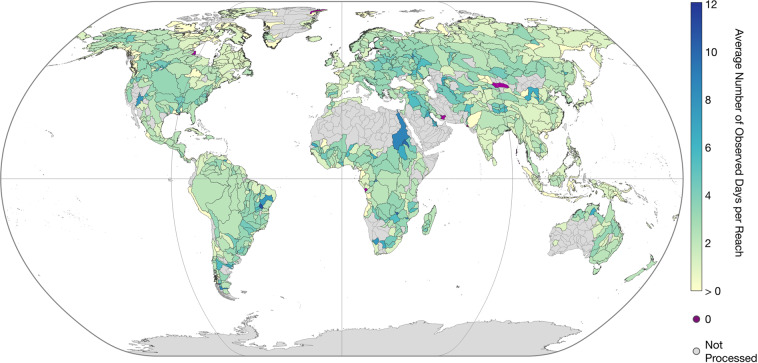
Average number of days with WSS results per Pfaffstetter Level 4 basin.

**Fig. 6:**
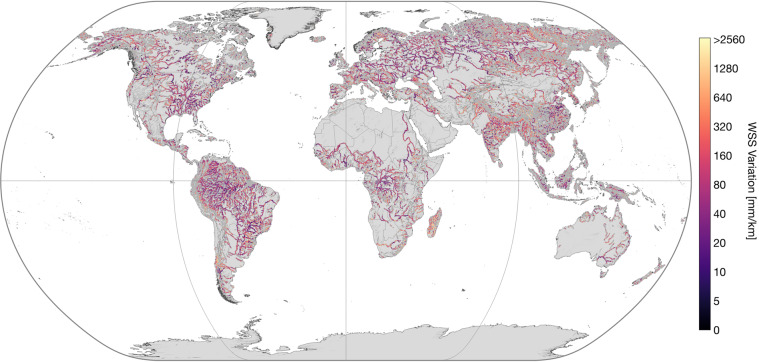
Combined water surface slope (WSS) variation. Only reaches with more than 3 days of record are shown.

**Fig. 7:**
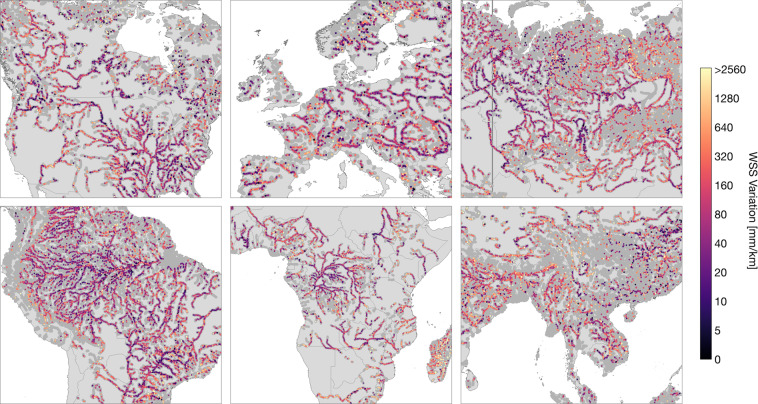
Combined water surface slope (WSS) variation details. Only reaches with more than 3 days of record are shown.

IRIS provides the number of days with WSS observations per reach by the parameter “*n_[across/along/combined]_slope*”. Figure 5 shows the average number of days with combined observations per basin. The detection of WSS changes over time is possible through the continuous addition of ICESat-2 observations. IRIS contains the minimum (“*min_[across/along/combined]_slope*”) and maximum (“*max_[across/along/combined]_slope*”) WSS obtained with the respective method per reach. The amplitude of WSS variations (“*max_combined_slope*” - “*min_combined_slope*”) for reaches with more than 3 days of WSS observations can be seen in Fig. 6. More detailed views of specific regions are provided in Fig. 7. The parameters “*min_date_[across/along/combined]_slope*” and “*max_date_[across/along/combined]_slope*” give the first and latest observation of the respective method per reach, and “*std_[across/along/combined]_slope*” provides the standard deviation of all respective WSS observations per reach.

## Technical Validation

The approach was validated at 815 reaches in a regional study^2^ with a median absolute error (MAE) of 23 mm/km for the “*avg_combined_slope*” compared to gauge data. The MAE for the “*avg_across_slope*” and “*avg_along_slope*” were 19 and 47 mm/km, respectively^2^. Although a global validation is not feasible, the overall robustness of the results can be inferred from Figs. 2, 3. Especially at the free-flowing Amazon and Congo Rivers, the WSS increases gradually in upstream direction. Discontinuities can be observed primarily in basins influenced by human intervention. For example, reservoirs in the western Mississippi River basin are apparent as single discontinuities with low WSS (cf. Fig. 3). In Figs. 6, 7, low variations of WSS can be observed along river main stems and regulated rivers. Large fluctuations, on the other hand, occur mainly at upstream reaches.

**Fig. 8:**
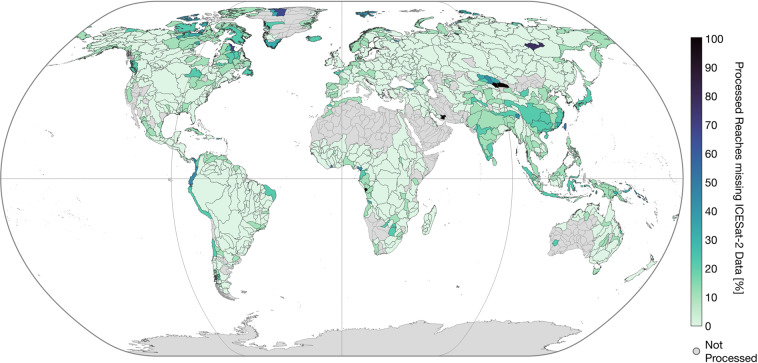
Percentage of processed reaches with missing ICESat-2 ATL13 data per Pfaffstetter level 4 basin.

Among others, high coverage can be achieved for Eastern Europe, Brazil, and the Nile River. Below-average coverage is apparent for parts of the Lena and Indus Rivers, Western Europe, East Asia, and the Pacific coast of South America. This is caused, among other factors, by missing ATL13 input data (cf. Figure 8). Overall, 16,754 (9.4%) of the processed reaches are not covered by ATL13 data.

**Fig. 9:**
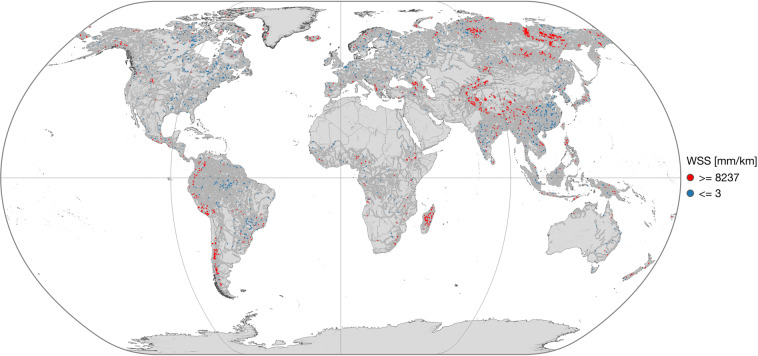
Averaged combined water surface slope (WSS) extremes. Reaches with an average WSS above the 99^th^ percentile (8,237 mm/km, red) and below the 1^st^ percentile (3 mm/km, blue).

**Fig. 10:**
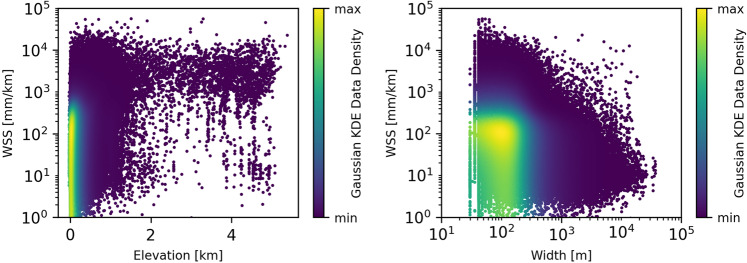
Averaged combined water surface slope (WSS) by reach elevation (left) and reach width (right).

Figure 9 displays extremely high (99th percentile: 8,237 mm/km) and low (1st percentile: 3 mm/km) “*avg_combined_slope*” WSS values. Except for some plateaus, low WSS values are located in low-lying regions. On the other hand, high WSS values are not limited to regions of high elevation as shown in Fig. 10 on the left. Figure 10 also provides the averaged combined WSS by reach width. With increasing width, the WSS tends towards 10 mm/km. Extremely high WSS are limited to narrow reaches. The data density in Fig. 10 indicates that the majority of the studied reaches are less than 300 m wide and situated lower than 200 m.

**Fig. 11:**
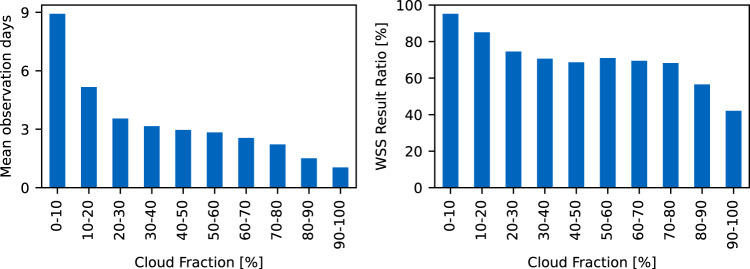
Mean number of observation days (left) and processed reaches with Water Surface Slope (WSS) results (right) by mean cloud fraction.

**Fig. 12:**
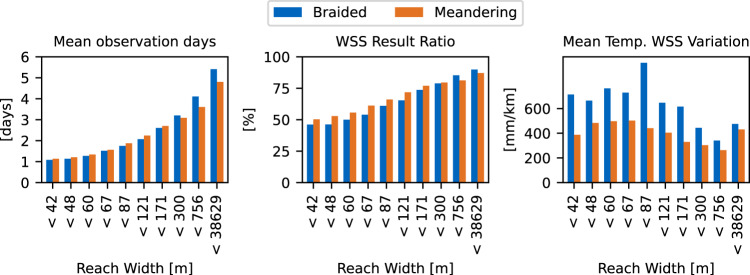
Mean number of observation days, WSS result ratio, and mean WSS variation per reach width grouped by 10% percentiles and river morphology.

In Fig. 11 the number of observed days and and the coverage with WSS results is compared with cloud coverage data from MODIS^28^ during the ICESat-2 period. Both values, especially the number of observed days, depend strongly on cloud coverage since the ATLAS lidar sensor cannot penetrate clouds. Additionally, Fig. 12 shows the number of observed days, the coverage with WSS results, and mean temporal WSS variation (“*max_combined_slope*” - “*min_combined_slope*”) per 10% reach width percentiles for meandering (*n* = 96,900) and braided (*n* = 24,683) rivers. For the morphological classification, the “Global River Morphology” (GRM) raster from the “Global Channel Belt” (GCB^29,30^) dataset is sampled along each reaches’ centerline, assigning the class with the highest summed probability. Figure 12 shows that with increasing width, more observations can be provided. The mean temporal WSS variation is significantly larger for braided rivers than for meandering rivers, especially at widths below 171 m.

**Fig. 13:**
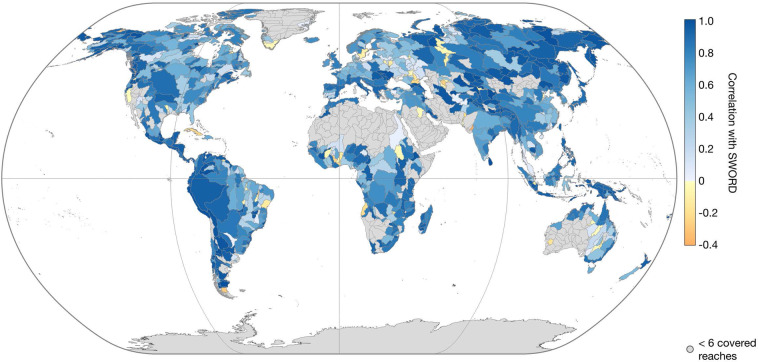
Correlation coefficient between IRIS average combined WSS and SWORD WSS from MERIT Hydro per Pfaffstetter level 4 basin enclosing more than 5 reaches covered with WSS results.

**Fig. 14:**
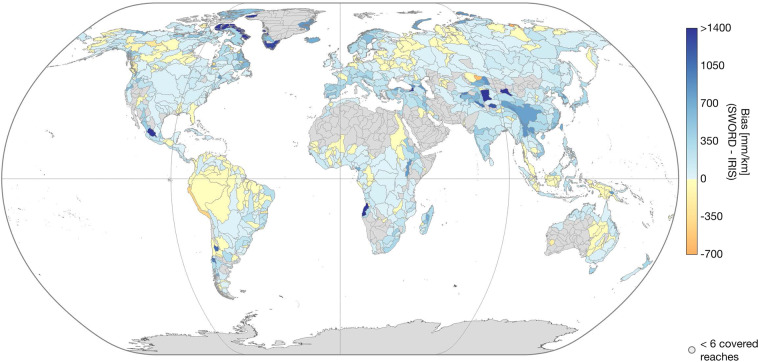
Bias (SWORD - IRIS) between IRIS average combined WSS and SWORD WSS from MERIT Hydro per Pfaffstetter level 4 basin enclosing more than 5 reaches covered with WSS results.

**Fig. 15:**
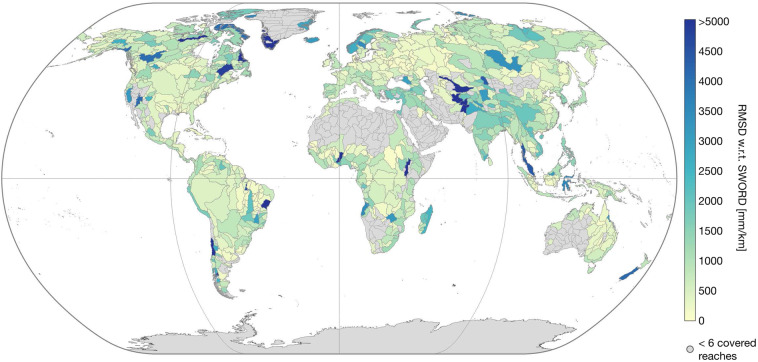
Root mean square deviation (RMSD) between IRIS average combined WSS and SWORD WSS from MERIT Hydro per Pfaffstetter level 4 basin enclosing more than 5 reaches covered with WSS results.

DEM data cannot be used to validate the accuracy of the results. However, for further analysis of the statistical soundness of the IRIS WSS, we compare the “*avg_combined_slope*” to the SWORD WSS derived from the MERIT Hydro DEM. Figures 13–15 show the correlation coefficient *r*, the bias (SWORD - IRIS), and the root mean square deviation (RMSD) between the two datasets per Pfaffstetter level 4 basin. Basins with less than six reaches covered by IRIS are not included because there is not enough data to make a meaningful comparison. On basin scale, *r* is greater than 0.50 for 580 (72%) out of the 808 basins with more than 5 covered reaches. Of the major river basins, only the Nile has a low correlation (0.03, cf. Figure 13). Over all IRIS and SWORD WSS, *r* is 0.73.

The bias ranges from −657 to 4,774 mm/km at the basin scale (with more than 5 reaches covered) with a mean of 149 mm/km. Thus, SWORD tends to have greater WSS than IRIS, especially in East Asia, while for almost the entire Amazon basin, the WSS results are greater in the IRIS dataset (cf. Figure 14). The overall RMSD after subtracting the bias between SWORD and IRIS is 1,514 mm/km, ranging from 12 to 19,994 mm/km at the basin scale (with more than 5 reaches covered) with a mean RMSD of 942 mm/km.

One reason for low correlations or large biases at the basin scale could be the gap between the acquisition time of the SWORD and IRIS input data, which spans 18 years in the case of SRTM and ICESat-2. During this time, significant hydraulic structures affecting the WSS may have been constructed and the river morphology may have changed. In addition, the bias between the two datasets is expected due to the better vertical accuracy of ICESat-2 compared to the MERIT DEM^31^ used to derive the SWORD WSS. The MERIT DEM shows significant elevation errors^21^ compared to the first ICESat mission in basins where large biases occur between IRIS and SWORD (e.g., in East Asia).

**Fig. 16:**
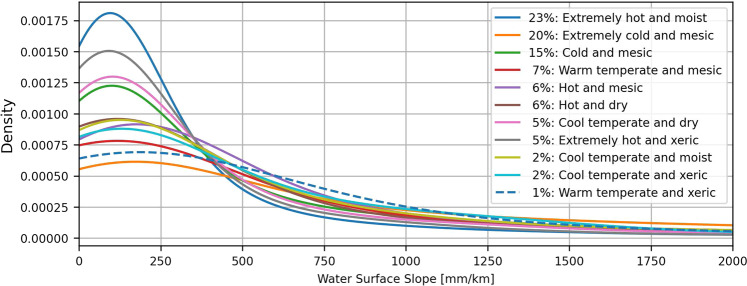
Probability density of the IRIS average combined WSS per climate zone.

**Fig. 17:**
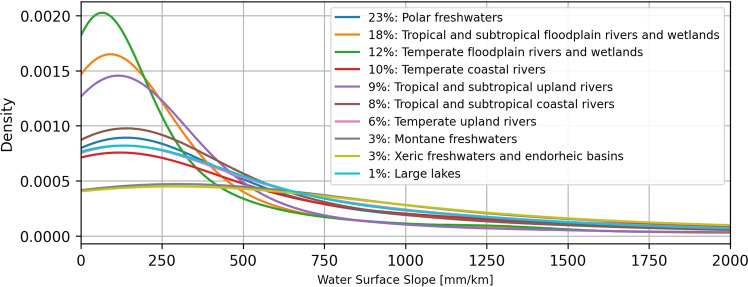
Probability density of the IRIS average combined WSS per freshwater habitat.

For an additional analysis of the statistical soundness, we classify the “*avg_combined_slope*” by different hydro-environmental classifications provided by HydroATLAS^32^ at the basin scale (BasinATLAS). Figures 16, 17 show the WSS probability density by different climate zones^33^ and freshwater habitats^34^, respectively. The legends listing the classes also show the relative contribution of each class to the total number of basins. Classes with a percentage of less than 1% are not shown.

## Usage Notes

Although IRIS contains separate values for each of the three approaches, the “*avg_combined_slope*” is likely to be appropriate for most use cases because it has the highest spatial and temporal coverage. Users should consider the number of days with WSS observations (“*n_[across/along/combined]_slope*”) when using the aggregated “*max_[across/along/combined]_slope*”, “*min_[across/along/combined]_slope*”, and “*std_[across/along/combined]_slope*” parameters. With fewer samples, these aggregated parameters get less significant or not meaningful at all. In this paper, only reaches with more than 3 days of WSS observations were used when plotting the parameters (i.e., Figs. 6, 7). IRIS will be updated progressively by adding future ICESat-2 cycles. This will provide increasing insight into the temporal variability of WSS. IRIS will also be updated with new versions of SWORD. Additionally, for future versions it is planned to include WSS uncertainty values derived from the confidence of fit or the WSE uncertainties for the along- and across-track method, respectively. Note, that the IRIS dataset is generated fully automatically and depends on the availability and quality of the ATL13 observations and flags, as well as the accuracy of the SWORD centerline, topology, and type parameters. Therefore, isolated outliers cannot be excluded, e.g., caused by missing dams in SWORD.

## Data Availability

A code example of IRIS is available at Zenodo^35^. The methodology is described in detail in our regional study^2^.
